# Draft Genome Sequences of Three Terrestrial Isoprene-Degrading *Rhodococcus* Strains

**DOI:** 10.1128/genomeA.01256-17

**Published:** 2017-11-09

**Authors:** Andrew T. Crombie, Helen Emery, Terry J. McGenity, J. Colin Murrell

**Affiliations:** a School of Environmental Sciences, University of East Anglia, Norwich, United Kingdom; b School of Biological Sciences, University of Essex, Wivenhoe Park, Colchester, Essex, United Kingdom

## Abstract

Isoprene is produced in abundance by plants and constitutes a carbon source for microbes. The genomes of three isoprene degraders isolated from tree leaves or soil from the campus of the University of East Anglia were sequenced. These high-GC-content isolates are actinobacteria belonging to the genus *Rhodococcus*.

## GENOME ANNOUNCEMENT

The emissions of isoprene to the atmosphere from terrestrial plants, principally trees, are similar in magnitude to those of methane (approximately 550 Tg per year). Some bacteria are capable of using isoprene as a sole source of carbon and energy, but their diversity and contribution to cycling of this climate-active trace gas have not been intensively studied until recently (1, 2). So far, genome sequences for a relatively small number of isoprene-degrading strains have been published (3–5).

*Rhodococcus* sp. strains ACPA1 and ACPA4 were isolated from the leaves of a white poplar tree (*Populus alba*) and *Rhodococcus* sp. strain ACS1 was isolated from soil in the vicinity of willow trees (*Salix fragilis*) located on the campus of the University of East Anglia, Norwich, United Kingdom. Isolates were grown in liquid culture supplied with isoprene, as described previously (3). Genomic DNA was extracted using a conventional phenol-chloroform method (3). For each strain, genomic DNA was sequenced by Edinburgh Genomics (Edinburgh, UK), following the construction of three libraries with inserts of 330, 550, and 4,500 bp, on an Illumina MiSeq instrument generating 300-nucleotide (nt) paired-end reads. Reads were trimmed using Cutadapt version 1.8.3 (6) using parameters -q 30 and -m 50, assembled using SPAdes version 3.7.0 (7) (removing contigs shorter than 200 bp), and annotated using the NCBI Prokaryotic Genome Annotation Pipeline (PGAP). The basic genome statistics are shown in Table 1.

**TABLE 1:** Genome statistics and accession numbers

Isolate	Genome size (Mbp)	Coverage (×)	No. of contigs	*N*_50_ (Mbp)	G+C content (%)	No. of rRNA operons	No. of tRNAs	No. of CDSs^a^	GenBank accession no.
ACPA1	10.06	238	47	1.38	66.9	1	68	9,193	NSDX00000000
ACPA4	7.07	296	9	5.07	61.6	3	55	6,473	NSDY00000000
ACS1	10.89	172	40	1.74	67.1	1	70	10,062	NSDZ00000000

a CDSs, coding sequences.

The large genome sizes (7 to 11 Mbp) are typical of metabolically versatile rhodococci (8), although the genome of *Rhodococcus* sp. ACPA4 is significantly smaller and of lower GC content than those of the other two strains. Based on analysis of the 16S rRNA genes, *Rhodococcus* sp. strains ACPA1 and ACPA4 are most closely related to the isoprene degraders *Rhodococcus opacus* PD630 (9) and *Rhodococcus* sp. strain AD45 (3), respectively, and *Rhodococcus* sp. strain ACS1 is related most closely to a non-isoprene-degrading *Rhodococcus koreensis* strain (10). All three genomes contain high-similarity homologues (>80% amino acid identity) of the isoprene metabolic genes described in *Rhodococcus* sp. AD45 (3, 11), including those encoding the soluble diiron center isoprene monooxygenase (*isoABCDEF*), glutathione-*S*-transferase (*isoI*), dehydrogenase (*isoH*), and genes for enzymes predicted to perform subsequent metabolic steps (*isoG* and *isoJ*). As in other isoprene degraders, *isoGHIJ* are duplicated nearby, while *Rhodococcus* sp. ACPA4 also contains a third copy of *isoH* and *isoJ*. The glutathione biosynthesis genes *gshA* and *gshB* are also present in all three strains, consistent with the observation that conjugation of isoprene epoxide with glutathione appears to be universal among isoprene degraders, despite the uncommon usage of this small thiol in Gram-positive bacteria (12, 13).

Interestingly, the genomes of *Rhodococcus* sp. strains ACPA1 and ACS1 contain an additional soluble diiron center monooxygenase in a different region of the genome, with high similarity (>90% amino acid identity) to propane monooxygenase from *Gordonia* TY-5 (14), indicative of the ability of many isoprene-degrading strains to grow on short-chain alkanes in addition to isoprene (5, 15).

These genome sequences extend the diversity of known *iso* genes and will enable the development of improved gene probes and molecular ecology methods for the detection of isoprene degraders in the environment.

### Accession number(s).

This whole-genome shotgun project has been deposited in DDBJ/ENA/GenBank under the accession numbers shown in Table 1. The versions described in this paper are the first versions.
